# Effectiveness of an Emotional Intelligence Intervention and Its Impact on Academic Performance in Spanish Pre-Adolescent Elementary Students: Results from the EDI Program

**DOI:** 10.3390/ijerph17207621

**Published:** 2020-10-19

**Authors:** María-José Cantero, Raquel Bañuls, Paz Viguer

**Affiliations:** 1 Developmental and Educational Psychology, University of Valencia, 46010 Valencia, Spain; maria.j.cantero@uv.es; 2 Edipsicólogos Center, 46970 Valencia, Spain; raquel@edipsicologos.com

**Keywords:** children health, emotional intelligence, school-based intervention, elementary school, academic performance

## Abstract

There is clear agreement about the importance of promoting emotional intelligence in school through programs integrated in the academic curriculum. The objective of this study is to analyze the impact of the EDI program on the emotional intelligence trait and on general academic performance, as well as mathematics and language performance. The participants were 5th grade elementary school students between 10 and 11 years old. A quasi-experimental repeated-measures design was used, with a comparison group and four assessment points. The results showed, on the one hand, the effectiveness of a two-year intervention in improving emotional intelligence and, on the other hand, its positive influence on academic performance in general, and specifically on mathematics and language performance. In the non-intervention group, general academic performance and language performance declined. The results are discussed, and recommendations are made for future interventions.

## 1. Introduction

Currently, there is a call for work on emotional intelligence (EI) at early ages because this type of intervention seems to improve EI while also positively affecting other factors in the school environment, including academic performance (AP). However, the data on this issue are inconclusive, basically due to the different EI conceptualizations on which the studies are based. According to the ability model, EI is a mental ability that unifies emotions and reasoning by facilitating more effective thinking, and it includes four skills: (a) perceiving, evaluating, and expressing emotions; (b) generating feelings that facilitate thinking; (c) understanding emotions; and (d) regulating emotions [1]. In addition, according to the trait EI model, EI is defined as a constellation of self-perceptions related to emotions at lower levels of personality hierarchies that integrates the affective aspects of personality [2,3]. Both models view EI as developing over time, and they propose that taking care of students’ emotional and social needs can lead to improvements, not only in EI, but also in numerous adaptive variables, such as psychological well-being, the quality of personal relationships, conflict resolution, and academic performance [4].

This study is based on the model by Bar-On [4], who, within the framework of the trait model, defines EI as a set of emotional and social competences related to understanding oneself and others effectively and relating to people and facing the demands, challenges, and daily pressures of the environment. For this author, the enabling influence of EI in an academic setting has traditionally been attributed to the students’ ability to function adequately in this complex socio-emotional environment. People with high emotional competence have better social skills, more stable long-term relationships, and better problem-solving skills. In addition, children with high emotional competence are better able to concentrate on problems and use problem-solving skills that increase their cognitive abilities. Schools that implement social and emotional learning programs report increases in academic success, higher quality relationships between teachers and students, and decreases in behavioral problems [5]. Durlak et al. [6] performed an important meta-analysis of 213 studies carried out in the US. The results indicated that interventions to improve social and emotional learning can be effective in improving social and emotional skills, attitudes, behavior, and AP. Our study is part of this field of research, and it analyzes the possibility of improving EI in pre-adolescent students through an intervention based on the Bar-On model, and the impact of this intervention on AP.

### 1.1. Emotional Intelligence and Academic Performance

The literature from the past two decades has paid increasing attention to EI as a potential predictor of better school adjustment throughout students’ development [7,8,9]. Costa and Faria [10] argued that emotional knowledge is related to better academic adjustment and student achievement, positive social behaviors, less distress, and better test and evaluation results. In addition, students with greater emotional competence have higher academic goals and grades, and they show better levels of self-discipline, motivation, stress regulation, work organization, and overall learning [11]. Petrides et al. [7] offer two explanations for the influence of trait EI on AP. First, high trait EI can have a psychologically stabilizing effect during the period before evaluations, although this differs depending on the student’s IQ. The effect of EI on AP occurs when demands tend to exceed the student’s intellectual resources, forcing students with a low IQ to resort to resources other than their cognitive ability to meet the demands. Second, trait EI can differentially affect performance by providing an advantage in academic subjects that involve affective-relational issues (for example, literature, and art). Moreover, Panayiotou et al. [12] recently presented an integrative model using a longitudinal sample of 9 to 12-year-old students in England. They found that students with greater social-emotional competence were reported to experience fewer mental health difficulties at Time 2, and this in turn predicted higher academic achievement at Time 3.

Nevertheless, research exploring the relationship between EI and AP has shown inconsistent results that may be due to the way AP is evaluated, differences in the characteristics of the sample (for example, sex, age, education level), and, especially, the different types of conceptualizations and measures of EI [10]. In this regard, based on the trait model, some studies report non-significant relationships [13,14,15]. However, the results of a meta-analytical review indicate the existence of a modest relationship between trait EI and AP [16]. In turn, Petrides et al. [7] examined the relationships between EI, cognitive ability, and AP in British secondary education, and they found that trait EI moderated the relationship between AP and cognitive ability. Furthermore, Qualter et al. [17], in a longitudinal study with British secondary school students, found that ability EI and trait EI at age 11 predicted AP at age 16. In a recent meta-analysis, MacCann et al., [18] examined the degree to which students’ EI is associated with academic performance, and they proposed that three mechanisms underlie the EI and AP link: regulating academic emotions, building social relationships at school, and the overlap between academic content and EI.

Most studies show an association between trait EI and AP in secondary school students and university students; however, this relationship has been studied less in pre-adolescents, and so it is necessary to confirm these results at the end of elementary education. In the meta-analytical review by Perera and Digiacomo [16], only six of the 48 samples studied were from elementary education. Observing the few studies that analyze the relationship between trait EI and AP in children and pre-adolescents, we find that they also confirm the relationship between these two constructs. The results of the study by Buenrostro-Guerrero, et al. [19] show positive correlations between trait EI and AP in Mexican pre-adolescents (11–12 years old). Along the same lines, Agnoli et al. [20] reported an interaction between trait EI and cognitive ability in predicting AP in a sample of Italian children (8–12 years old). In the Spanish context, two studies reported a positive correlation between trait EI and AP in pre-adolescents (11–12 years old) [21,22].

### 1.2. Emotional Intelligence and Academic Performance in Specific Subjects

As we have seen, some studies have analyzed the relationship between trait EI and AP, but few have separately analyzed EI’s impact on performance in subjects such as language, mathematics, science, etc. The few studies carried out in secondary education or at the end of elementary education suggest that the effects of trait EI may vary across educational levels, age, and academic subjects.

In the study by Petrides et al. [7], trait EI was separately associated with different subjects. EI did not have a considerable influence on mathematics performance (MP) or science performance, but it moderated the effect of IQ on language performance (LP) and overall academic performance. Mavroveli et al. [23] found that trait EI showed statistically significant associations with English (LP) and math scores, but when age and non-verbal intelligence were held constant, these correlations lost their significance. Furthermore, Agnoli et al. [20] found an interaction between trait EI and cognitive ability in predicting AP. Specifically, trait EI was positively associated with better LP in children with low or medium cognitive ability, but not in students with a high cognitive level. No moderating effect of trait EI was found in the relationship between cognitive ability and MP. However, other studies have found a relationship between trait EI and MP [24,25,26]. These findings suggest that trait EI improves MP, regardless of cognitive ability.

Whereas in language a high cognitive ability may be sufficient to meet the demands of the subject, the greater complexity of mathematics may require additional resources [27]. In addition, high EI can help to cope with the anxiety that, according to Ashcraft [28], is usually associated with mathematics. In Spain, two studies analyzed the predictive capacity of trait EI on AP. In the Belmonte study [29], trait EI was a significant predictor of AP in the scientific, linguistic, and technological fields. Furthermore, Pérez-González et al. [30] found that both ability EI and trait EI had positive and significant correlations with AP in different subjects, and they were significant predictors of overall performance in secondary school.

### 1.3. Our Study

Most studies focus on analyzing the relationship between trait EI and AP, but few studies analyze the influence of an EI intervention on AP in Europe, and specifically in the Spanish context. This study, to our knowledge, is the first to use a rigorous EI intervention program with Spanish elementary school students (10–12 years old). The main objective of the study is to analyze the impact of the EDI program: Would you like to travel around the planet of emotions? on EI, general academic performance, and mathematics and language performance in 5th and 6th grade elementary school students. Thus, we hypothesize the following: (1) the program will significantly increase the participants’ EI, whereas the students without the intervention will show no improvement; (2) the intervention will improve the students’ mathematics, language, and general academic performance, whereas without the intervention, performance will not change. A quasi-experimental design with an intervention group and a comparison group was used.

## 2. Materials and Methods 

### 2.1. Participants

A total of 245 pupils in fifth grade of elementary school initiated the study. Contact was made with all the public schools in two neighboring Spanish municipalities from the same Education Area (Valencia, Spain) with the same curriculum. Both municipalities belonged to the metropolitan area of Valencia city and had a total population of about 20,000 inhabitants. Families from these public schools had similar socio-demographic characteristics and socio-economic status. Initially, three schools in the first municipality decided to participate, and two in the second one. Students from the first municipality were assigned to the intervention group, and those from the second municipality were assigned to the comparison group. This decision was made at the request of the educational authorities, so that the intervention condition could be given between municipalities, but not within municipalities. Unfortunately, one of the schools that participated in the comparison group decided to drop out of the study after the first assessment (63 students). The final sample consisted of 182 students; 98 were boys (53.8%), and their ages ranged from 10 to 11 years (M = 10.32; SD = 0.47). Regarding their nationalities, 89.6% were Spanish, and 10.4% were immigrants. In addition, 23.1% had learning disabilities. A total of 136 participants (74.7%) participated in the intervention group, and 46 (25.3%) in the comparison group. To confirm the homogeneity of the intervention and comparison groups, the sociodemographic data obtained in the initial evaluation were compared. The homogeneity tests revealed no significant differences between the groups: age (*t* (180) = − 0.12, *p* = 0.90), gender (χ^2^ (1, *N* = 182) = 0.01, *p* = 0.94), nationality (χ^2^ (1, *N* = 182) = 1.50, *p* = 0.22), and learning disabilities (χ^2^ (1, *N* = 182) = 0.31, *p* = 0.575).

### 2.2. Procedure and Intervention Design

This research complies with the ethical principles of the Declaration of Helsinki and the code of ethics of the Spanish Association of Psychologists, and the principles of confidentiality in the treatment of minors were guaranteed in accordance with current Spanish data protection laws (LO 3/2018 of 5 December). Various informative meetings were held with the selected schools to explain the objectives and methodology of the EDI program. School administrators, teachers, and families were informed about the study and gave their informed consent to participate. The students were informed that their participation in the study would be voluntary, anonymous, and confidential. None of the students refused to participate.

The EDI program is a school-based emotional intelligence program for children between 10 and 12 years old designed in Spain. In this study, the program was conducted by three trained psychologists, and the group’s teacher attended the sessions. The program was implemented over the course of two academic years, and it provided information about emotions and trained participants to apply them in everyday situations. The EDI program is structured as a journey through the world of emotions with EDI, a comic character who comes from the planet of emotions and feelings (Emotiko planet) and guides the children through the main activities of the program. We use a comprehensive approach that includes experiential methodology with psychophysical, psychodrama, and systemic techniques, as well as some cognitive-behavioral techniques. The structure of the intervention was based on techniques such as film forum, storytelling, drama, group discussion, case studies, relaxation, and music therapy. We also used techniques such as empathic stimulation, active listening, positive reinforcement, and modeling to strengthen learning. The program design was based on the Bar-On model and organized into four modules: (1) intrapersonal skills, whose objective was for students to know and understand their own emotions and those of others; (2) interpersonal skills, which sought to develop greater competence in their social relations by working on assertiveness and empathy; (3) emotion regulation, whose objective was for children to learn to manage emotions and develop tolerance to frustration and stress; and (4) general mood, which sought to promote a positive affective mood in the children. To enhance the transfer effect of the intervention, we focused on action-oriented exercises and the participants’ personal experiences, and we developed reality-based training materials.

The participants in the intervention group received the sessions of the EDI program over the course of two academic years during homeroom time. In each academic year, the intervention took place for sixteen weeks, with a one-week interval between sessions. During the same class period, participants in the comparison group received the usual homeroom lessons about classroom routines, coordination with students’ families, and study techniques. All participants were assessed at four points in time: (T1) Time 1, at the beginning of 5th grade and before the first intervention training (baseline); (T2) Time 2, at the end of the first intervention session; (T3) Time 3, follow-up evaluation at the beginning of 6th grade and before the start of the second intervention training; (T4) Time 4, at the end of 6th grade, coinciding with the end of the second intervention session. Six months elapsed between the assessment points.

### 2.3. Measures

Emotional intelligence was assessed with the Emotional Quotient Inventory (EQ-i: YV) [31] in its Spanish adaptation [18]. It is a self-report measure that evaluates the EI of children and adolescents between 7 and 18 years old. It consists of 60 items rated on a 4-point Likert response scale (1 = never happens to me to 4 = always happens to me). The instrument offers a total EI score based on scores obtained on the following scales: intrapersonal intelligence; interpersonal intelligence; stress management, and adaptability. A high score on the total scale indicates a high level of emotional competence. In our study, Cronbach’s alpha was 0.81 in the initial evaluation (T1), 0.86 in T2, 0.86 in T3, and 0.89 in T4.

Three measures of Academic Performance variable were considered: general academic performance (AP), mathematics performance (MP), and language performance (LP). AP was evaluated with the average grade in all subjects (from 0 to 10). The MP and LP were evaluated with the grade obtained in the mathematics and Spanish language subjects, respectively, evaluated from 0 to 10 points. These variables were recorded at the end of the first and third trimesters of 5th and 6th grades, coinciding with the beginning and end of the two-year intervention.

### 2.4. Analytic Plan

A quasi-experimental repeated-measures design was used, with a comparison group and four assessment points. Four mixed analyses of variance were carried out, considering group (intervention-comparison) as between-subject factor and time (T1–T4) as within-subject factor for the emotional intelligence, general academic performance, mathematics performance, and language performance variables. The effect size was calculated using partial eta squared $\eta_{p}^{2}$ and Cohen’s *d*. Post-hoc tests were performed using the Bonferroni adjustment for multiple comparisons. The statistical program SPSS version 24 (IBM, Armonk, NY, USA) was used to analyze the data.

## 3. Results

The means and standard error for emotional intelligence and the three performance variables in the intervention and comparison groups for each measurement time can be found in Table 1.

### 3.1. Emotional Intelligence

All bivariate correlations between the EI scores at different time points were positive and statistically significant in both groups (*p* < 0.01). In the intervention group, bivariate correlations ranged from 0.48 to 0.84; and in the comparison group, they ranged between 0.62 to 0.79.

Univariate analyses showed a significant principal effect of the group-time interaction, *F* (3, 540) = 13.59, *p* < 0.001, $\eta_{p}^{2}$ = 0.07. The study of the between-group simple effects confirmed that the intervention group and the comparison group did not differ significantly in their EI scores at the first measurement time, *F* (1, 180) = 0.26, *p* = 0.61, $\eta_{p}^{2}$ = 0.001, thus showing homogeneity in EI before the intervention. Significant differences in EI were obtained between the two groups in T2, *F* (1, 180) = 13.24, *p* < 0.001, $\eta_{p}^{2}$ = 0.07; in T3, *F* (1, 180) = 12.71, *p* < 0.001, $\eta_{p}^{2}$ = 0.07, and in T4, *F* (1, 180) = 57.24, *p* < 0.001, $\eta_{p}^{2}$ = 0.24, with higher EI scores in the intervention group (see Table 1).

**Figure 1:**
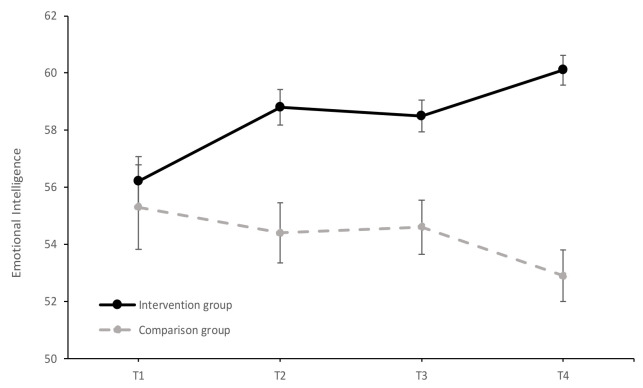
Evolution of the average EI scores (±SEM) in the intervention and comparison groups over time (T1 = baseline; T2 = at the end of the first year of intervention; T3 = follow-up after the first year of intervention; T4 = at the end of the second year of intervention).

Figure 1 shows the results of the study of the evolution of EI in the intervention and comparison groups over time (T1–T4). The simple multivariate effects of the time variable were significant in both the intervention group (Wilks λ = 0.73, *F* (3, 178) = 22.43, *p* < 0.001, $\eta_{p}^{2}$ = 0.27) and the comparison group (Wilks λ = 0.95, *F* (3, 178) = 3.24, *p* < 0.05, $\eta_{p}^{2}$ = 0.05). In the intervention group, there was a significant increase in scores from T1 to T2 (mean difference = 2.63, *SE* = 0.61, *p* < 0.001, *d* = 0.32, 95% CI [1.01, 4.25]), from T1 to T3 (mean difference = 2.31, *SE* = 0.67, *p* < 0.01, *d* = 0.28, 95% CI [0.52, 4.10]), and from T1 to T4 (mean difference = 4.62, *SE* = 0.74, *p* < 0.001, *d* = 0.57, 95% CI [2.66, 6.59]). Likewise, a significant increase in the EI score was obtained from T2 to T4 (mean difference = 2.00, *SE* = 0.46, *p* < 0.001, *d* = 0.32, 95% CI [0.76, 3.23]) and from T3 to T4 (mean difference = 2.31, *SE* = 0.32, *p* < 0.001, *d* = 0.38, 95% CI [1.47, 3.16]). Regarding the comparison group, a significant decrease in the EI scores was observed. This decrease occurred in the scores obtained between T3 and T4 (mean difference = −1.67, *SE* = 0.55, *p* < 0.05, *d* = −0.25, 95% CI [−3.12, −0.21]). These results suggest a significant increase in the EI scores in the intervention group over the two years of implementation of the EDI program, and a significant decrease in EI in the non-intervention group.

### 3.2. Academic Performance: General, Mathematics, and Language.

Bivariate correlations between the mathematics and language performance for T1–T4 were 0.71 (*p* < 0.01), 0.64 (*p* < 0.01), 0.75 (*p* < 0.01), and 0.65 (*p* < 0.01) for the intervention group, and 0.50 (*p* < 0.01), 0.36 (*p* < 0.05), 0.80 (*p* < 0.01), and 0.52 (*p* < 0.01) for the comparison group.

**Figure 2:**
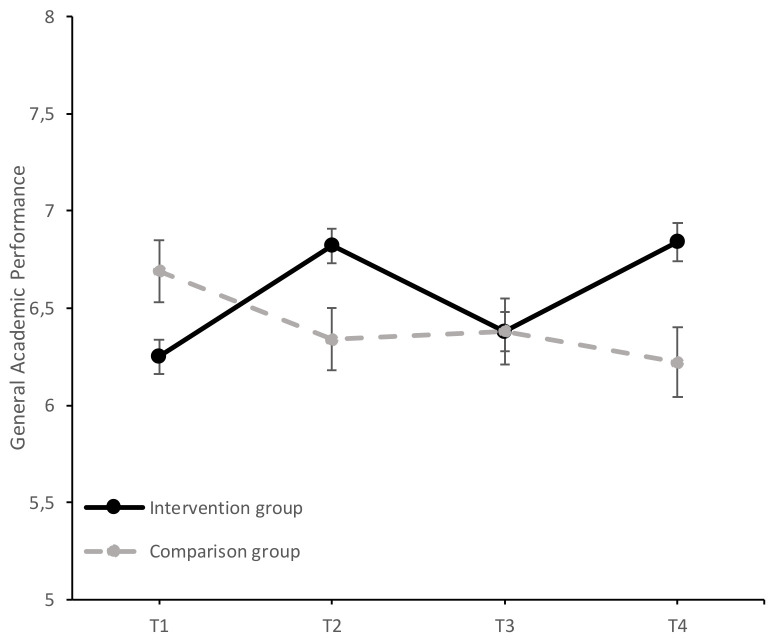
Evolution of the average AP scores (±SEM) in the intervention and comparison groups over time (T1 = baseline; T2 = at the end of the first year of intervention; T3 = follow-up after the first year of intervention; T4 = at the end of the second year of intervention).

**Figure 3:**
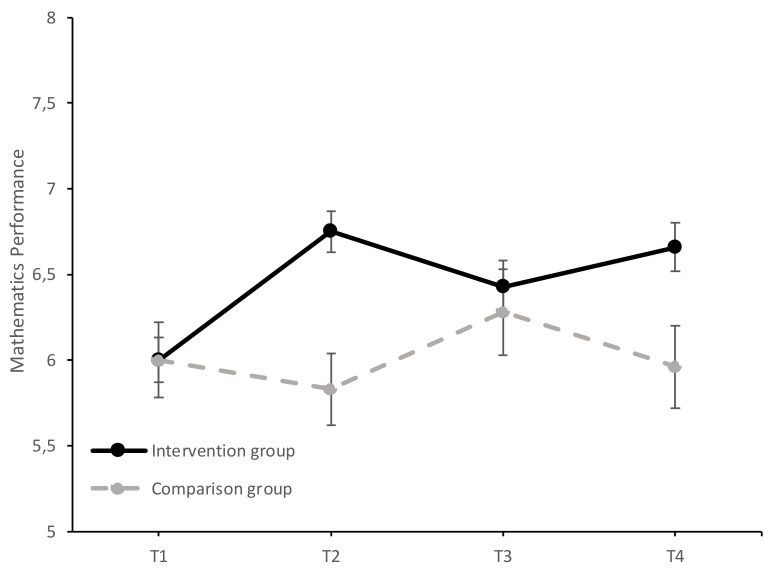
Evolution of the average MP scores (±SEM) in the intervention and comparison groups over time (T1 = baseline; T2 = at the end of the first year of intervention; T3 = follow-up after the first year of intervention; T4 = at the end of the second year of intervention).

**Figure 4:**
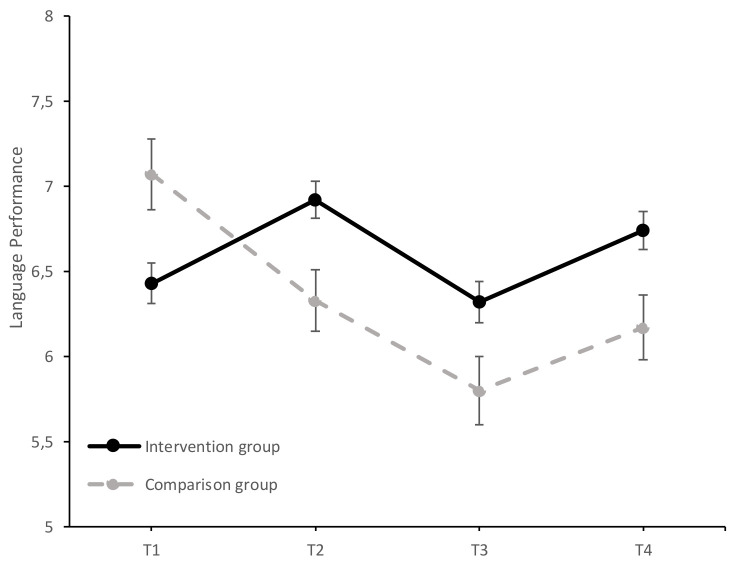
Evolution of the average LP scores (±SEM) in the intervention and comparison groups over time (T1 = baseline; T2 = at the end of the first year of intervention; T3 = follow-up after the first year of intervention; T4 = at the end of the second year of intervention).

Univariate analyses showed a significant principal effect of the time-group interaction on overall performance, *F* (3, 540) = 23.44, *p* < 0.001, $\eta_{p}^{2}$ = 0.12, mathematics performance *F* (3, 540) = 6.26, *p* < 0.001, $\eta_{p}^{2}$ = 0.03, and language performance, *F* (3, 540) = 15.92, *p* < 0.001, $\eta_{p}^{2}$ = 0.08. The results of the study of the evolution of AP, MP, and LP in both groups over time (T1–T4) can be found in Figure 2, Figure 3 and Figure 4. 

#### 3.2.1. General Academic Performance

The study of simple effects between groups confirmed that the intervention and comparison groups differed significantly on their AP scores, *F* (1, 180) = 5.81, *p =* 0.017, $\eta_{p}^{2}$ = 0.03, before the intervention (T1), with the comparison group showing better performance. After the first year of intervention (T2), significant differences were observed, *F* (1, 180) = 6.78, *p =* 0.01, $\eta_{p}^{2}$ = 0.04, but in this case in favor of the intervention group. In the follow-up measure (T3), the two groups matched their AP scores, *F* (1, 180) = 0.00, *p =* 0.99, $\eta_{p}^{2}$ = 0.00. After the second year of intervention (T4), the intervention group obtained a significantly higher performance score than the comparison group, *F* (1, 180) = 8.94, *p* < 0.01, $\eta_{p}^{2}$ = 0.05. This difference in performance in favor of the intervention group is especially relevant considering the non-homogeneity of the two groups at the beginning of the intervention, with the intervention group showing lower initial performance. However, it should also be noted that this better final performance could lead us to suspect that there had been a regression to the mean (regression artifact). The intervention group was lower at baseline and, hence, more susceptible to gains, even if the treatment has no effect.

The simple multivariate effects of the time variable were significant in both the intervention group (Wilks λ = 0.40, *F* (3, 178) = 90.75, *p* < 0.001, $\eta_{p}^{2}$ = 0.61) and the comparison group (Wilks λ = 0.85, *F* (3, 178) = 10.39, *p* < 0.001, $\eta_{p}^{2}$ = 0.15). In the intervention group, there was a significant increase in scores from T1 to T2 (mean difference = 0.57, *SE* = 0.04, *p* < 0.001, *d* = 0.51, 95% CI [0.47, 0.67]) and from T1 to T4 (mean difference = 0.59, *SE* = 0.09, *p* < 0.001, *d* = 0.49, 95% CI [0.36, 0.82]). However, there was a significant decrease in the AP score from T2 to T3 (mean difference = −0.43, *SE* = 0.07, *p* < 0.001, *d* = −0.39, 95% CI [−0.62, −0.25]), again significantly increasing from T3 to T4 (mean difference = 0.45, *SE* = 0.06, *p* < 0.001, *d* = 0.39, 95% CI [0.28, 0.62]). The effect size was moderate for the comparison between T1 and T2 and between T3 and T4, coinciding with the two intervention periods. In the comparison group, there was a significant decrease in AP scores from T1 to T2 (mean difference = −0.35, *SE* = 0.06, *p* < 0.001, *d* = −0.41, 95% CI [−0.52, −0.17]) and from T1 to T4 (mean difference = −0.47, *SE* = 0.15, *p* < 0.05, *d* = −0.49, 95% CI [−0.87, −0.07]). These results suggest that the AP scores significantly increased in the intervention group during the two program implementation periods (T1–T2 and T3–T4), whereas the comparison group’s scores decreased progressively (T1–T2 and T1–T4).

#### 3.2.2. Mathematics Performance

The study of simple effects between groups confirmed that the intervention and comparison groups did not differ significantly in their MP scores in T1, *F* (1, 180) = 0.00, *p =* 1.00, $\eta_{p}^{2}$ = 0.00, and so they were homogeneous before the intervention. Significant differences were observed in favor of the intervention group in T2, *F* (1, 180) = 15.00, *p* < 0.001, $\eta_{p}^{2}$ = 0.08. No significant differences were obtained between the two groups in T3, *F* (1, 180) = 0.28, *p =* 0.60, $\eta_{p}^{2}$ = 0.00, and in T4 the intervention group again obtained a significantly higher score than the comparison group, *F* (1, 180) = 6.41, *p* < 0.01, $\eta_{p}^{2}$ = 0.03.

The simple multivariate effects of the time variable were significant in the intervention group (Wilks λ = 0.71, *F* (3, 178) = 24.62, *p* < 0.001, $\eta_{p}^{2}$ = 0.29), but not in the comparison group (Wilks λ = 0.97, *F* (3, 178) = 1.88, *p* = 0.14, $\eta_{p}^{2}$ = 0.03). In the intervention group, there was a significant increase in scores from T1 to T2 (mean difference = 0.75, *SE* = 0.09, *p* < 0.001, *d* = 0.52, 95% CI [0.52, 0.98]), from T1 to T3 (mean difference = 0.43, *SE* = 0.14, *p* = 0.01, *d* = 0.28, 95% CI [0.07, 0.80]), and from T1 to T4 (mean difference = 0.66, *SE* = 0.15, *p* < 0.001, *d* = 0.42, 95% CI [0.27, 1.06]). This result indicates that the first year of intervention of the EDI program significantly increased MP (T1–T2), and the second year of program implementation (T3–T4) maintained the increase obtained. In contrast, the group with no intervention did not significantly change their MP scores over time.

#### 3.2.3. Language Performance

The study of simple effects between groups confirmed that the intervention and comparison groups differed significantly in their language performance scores, *F* (1, 180) = 7.13, *p =* 0.008, $\eta_{p}^{2}$ = 0.04, before the intervention (T1), with the comparison group showing higher performance. In T2, significant differences were observed, *F* (1, 180) = 7.82, *p =* 0.006, $\eta_{p}^{2}$ = 0.04, but in this case in favor of the intervention group. In T3, *F* (1, 180) = 4.90, *p =* 0.028, $\eta_{p}^{2}$ = 0.03, and T4, *F* (1, 180) = 6.49, *p =* 0.012, $\eta_{p}^{2}$ = 0.04, the two groups continued to differ in their scores, in favor of the intervention group. This better performance of the intervention group after the two-year program implementation is especially relevant considering the non-homogeneity of the two groups at the beginning of the intervention, when the intervention group had lower initial performance.

The simple multivariate effects of the time variable were significant in both the intervention group (Wilks λ = 0.76, *F* (3, 178) = 19.21, *p* < 0.001, $\eta_{p}^{2}$ = 0.25) and the comparison group (Wilks λ = 0.80, *F* (3, 178) = 15.13, *p* < 0.001, $\eta_{p}^{2}$ = 0.20). In the intervention group, there was a significant increase in scores from T1 to T2 (mean difference = 0.49, *SE* = 0.08, *p* < 0.001, *d* = 0.36, 95% CI [0.25, 0.73]). However, there was a significant decrease in scores from T2 to T3 (mean difference = −0.60, *SE* = 0.11, *p* < 0.001, *d* = −0.44, 95% CI −0.90, −0.31]), with the scores at follow-up matching those obtained in the initial evaluation (mean difference = −0.11, *SE* = 0.12, *p* = 1.0, *d* = −0.01, 95% CI [−0.42, 0.20]). After the second year of intervention (T3-T4), the scores again increased significantly (mean difference = 0.43, *SE* = 0.09, *p* < 0.001, *d* = 0.30, 95% CI [0.19, 0.67]). Despite these oscillations, the LP increased significantly from the initial evaluation (T1) to the final evaluation after the second year (T4) (mean difference 0.32, *SE* = 0.12, *p* < 0.04, *d* = 0.22, 95% CI [0.01, 0.63]), with a small effect size.

In the comparison group, there was a significant decrease in the LP scores from T1 to T2 (mean difference = −0.74, *SE* = 0.16, *p* < 0.001, *d* = −0.64, 95% CI [−1.15, −0.33]), from T1 to T3 (mean difference = −1.26, *SE* = 0.20, *p* < 0.001, *d* = −1.03, 95% CI [−1.79, −0.74]), from T1 to T4 (mean difference = −0.89, *SE* = 0.20, *p* < 0.001, *d* = 0.77, 95% CI [−1.42, −0.36]), and from T2 to T3 (mean difference = −0.52, *SE* = 0.19, *p* = 0.04, *d* = −0.46, 95% CI [−1.03, −0.01]). These results suggest that the LP scores of the intervention group significantly increased during the program’s implementation, whereas the non-intervention group’s scores decreased progressively (T1–T2 and T1–T4), with the effect sizes of the differences being moderate and high.

## 4. Discussion

The first aim of this study was to analyze the effectiveness of the EDI program in enhancing EI in 5th and 6th grade elementary school children. The results highlight the effectiveness of our intervention in improving EI over time, thus confirming our first hypothesis. The first year of intervention improved the EI results, and this increase remained stable six months later, which meant that at the beginning of the second year of intervention, students were starting at the level achieved in the previous year. The second year of intervention continued to significantly improve EI. However, in the group without the intervention, EI remained stable during the first year and decreased significantly during the second. The results confirm the significant improvement in EI as a result of the intervention, demonstrating the effectiveness of the EDI program, which is consistent with what previous studies have suggested [32]. In addition, it should be noted that the differences between the intervention group and the non-intervention group were greater after the second year. This result indicates that the first year of intervention improves EI significantly, but a long-term intervention would maintain these levels and even develop them further. Therefore, this study provides clear evidence of the need to apply programs to enhance EI during several academic school years. This contribution coincides with the recommendation by Zeidner et al. [33] to develop intervention plans over several years, in order to provide repeated opportunities for students to discover more about their emotional competencies as they develop. In addition, our study points out the importance of intervening, especially in 6th grade, given that the results reflect a clear decrease in EI without the intervention during this academic year. Pre-adolescence is a stage involving great developmental changes that can have a negative impact on students’ emotional competencies. Therefore, an intervention in this time period aids the transition to adolescence by encouraging young people’s positive development. Moreover, considering that previous studies suggest that adolescents with high EI deal with the transition to compulsory secondary education better [34], the importance of carrying out rigorous interventions during the last years of elementary education is undeniable.

Second, this study aims to provide evidence about the effectiveness of the EDI program in improving students’ overall general academic performance (AP). The results confirm our second hypothesis. During the period of the program’s implementation, the AP of the students in the intervention group improved, unlike what occurred in the group without the intervention. However, the improvement achieved during the first year declines to the initial levels in the follow-up evaluation at the beginning of 6th grade, matching the scores of the group without the intervention. The second year of intervention increases the AP again, reaching the scores obtained at the end of the first year of intervention. Thus, the EI intervention positively influences performance during the intervention period. This result again reveals the importance of long-lasting interventions, given their impact on AP. Our results, in line with the few previous studies [16,19,20,21,22], provide new evidence about the relationship between trait EI and AP in pre-adolescents, while also showing the benefits of the trait EI intervention for AP.

Regarding the impact of the EI intervention on academic performance in specific curricular areas, the hypothesis is also confirmed, although the trend observed in the second year of intervention was different for MP and LP. In the case of MP, the first year of intervention improved the results, and this increase remained stable after 6 months. There was no further improvement in the second year, but the progress was maintained. The non-intervention group did not change their MP scores. This result supports the idea that EI intervention can help students to cope with the anxiety caused by mathematics [28] because the complexity of this subject causes students to draw on their emotional competencies to address it [25,26]. Regarding LP, as with MP, the first year of intervention improved the LP results, but this improvement did not remain stable after six months. The second year of intervention had a positive impact on LP, increasing it to the levels obtained at the end of the first year of intervention. In contrast, without the intervention, LP decreased progressively. These data support the idea that EI benefits school subjects that involve relational affective issues, such as language [7,20,29], especially considering that, without the intervention, there is a significant decrease in performance in this area.

Finally, some limitations should be addressed. As a first study limitation, we can mention the non-homogeneity of the two groups in general academic performance and LP, in favor of the comparison group. However, the two groups were homogeneous in terms of EI. Despite these limitations in AP and LP, the intervention managed to reverse the initial trend, with the intervention group achieving better final performance, even though it started from a lower initial level. Furthermore, it was not possible to match the number of participants in the two groups due to the difficulty of carrying out a long-term follow-up of the students without the intervention. Other limitations that are difficult to address are related to measurement error. We could question whether the average grade across four time points has been rated on the same metric/scale. Moreover, one potential source of error would be different teachers (who assign grades) or variations in grading criteria across the years.

Suggested future lines of research include, on the one hand, expanding the evaluation instruments by combining self-reports with performance measures, in order to complement the students’ self-perception with their performance on specific tasks. On the other hand, considering that in previous studies the relationship between trait EI and AP was modulated by cognitive ability [6,17], this variable should be controlled in future applications of the program. Moreover, further studies are needed to understand the generalizability of these results across different ages and cultures. New longitudinal interventions are currently being carried out using the EDI program with children between 8 and 10 years old. Our long-term goal is to create new versions of the program for children at all elementary levels and conduct new studies that reveal the effectiveness of the intervention and its impact on academic performance and mental health.

## 5. Conclusions

The article addresses the timely topic of an emotional intelligence intervention program (EDI) and its impact on academic performance in Spanish pre-adolescent elementary students. EI plays a vital role in many aspects of life, especially in the educational field, and intervention programs should be applied from a young age to improve emotional intelligence and achieve better academic results with less stress. In this framework, the two main contributions of this study are: (1) the confirmation of the effectiveness of a long-term intervention to improve EI in pre-adolescents, given that pre-adolescence is a relevant stage in which adequate socio-emotional development allows a smoother transition to adolescence; and (2) the contribution of new evidence about the positive influence of EI on AP, not only at a general level, but also in specific subjects such as mathematics and language. However, taking into account that this positive influence only remains stable during the intervention, new studies are required to analyze the stability of the change in academic performance. Finally, the findings of this study have some implications for educational policy. Future guidelines can recommend the implementation of the EI intervention in schools, especially with pre-adolescents. These interventions would be useful for promoting children’s well-being and mental health.

## Figures and Tables

**Table 1:** Mean and standard error (SEM) for the Emotional Intelligence and Academic Performance variables at T1-T4, *F* multivariate and post hoc comparisons.

	Group	T1	T2	T3	T4	*F* (3, 178), *p*	*Post hoc*
Emotional Intelligence	Intervention	56.18 (0.86)	58.81 (0.61)	58.49 (0.55)	60.81 (0.52)	22.4 *p* < 0.001	T1 < T2 *** T1 < T3 ** T1 < T4 *** T2 < T4 *** T3 < T4 ***
	Comparison	55.31 (1.48)	54.41 (1.05)	54.61 (0.94)	52.94 (0.90)	3.2 *p* = 0.02	T3 > T4 *
General Academic Performance	Intervention	6.25 (0.09)	6.82 (0.09)	6.38 (0.10)	6.84 (0.10)	90.8 *p* < 0.001	T1 < T2 * T1 < T4 *** T2 > T3 *** T3 < T4 ***
	Comparison	6.69 (0.16)	6.34 (0.16)	6.38 (0.17)	6.22 (0.18)	10.4 *p* < 0.001	T1 > T2 *** T1 > T4*
Mathematics Performance	Intervention	6.00 (0.13)	6.75 (0.12)	6.43 (0.15)	6.66 (0.14)	24.6 *p* < 0.001	T1 < T2 *** T1 < T3 * T1 < T4 ***
	Comparison	6.00 (0.22)	5.83 (0.21)	6.28 (0.25)	5.96 (0.24)	1.9 *p* = 0.14	N/A
Language Performance	Intervention	6.43 (0.12)	6.92 (0.11)	6.32 (0.12)	6.74 (0.11)	19.2 *p* < 0.001	T1 < T2 *** T1 < T4 * T2 > T3 *** T3 < T4 ***
	Comparison	7.07 (0.21)	6.33 (0.18)	5.80 (0.20)	6.17 (0.19)	15.1 *p* < 0.001	T1 > T2 *** T1 > T3 *** T1 > T4 *** T2 > T3 *

Note. T1 = baseline; T2 = at the end of the first year of intervention; T3 = follow-up after the first year of intervention; T4 = at the end of the second year of intervention. *F*: simple multivariate effect of time in intervention and comparison groups. N/A not applicable, * *p* < 0.05; ** *p* < 0.01, *** *p* < 0.001.

## References

[1] Mayer J.D., Salovey P., Caruso D.R. (2004). Emotional intelligence: Theory, findings, and implications. Psychol Inq..

[2] Petrides K.V., Furnham A., Mavroveli S. (2007). Trait emotional intelligence: Moving forward in the field of EI. Emot. Intell. Knowns Unkn..

[3] Siegling A.B., Furnham A., Petrides K.V. (2015). Trait emotional intelligence and personality: Gender-invariant linkages across different measures of the Big Five. J. Psychoeduc. Assess..

[4] Bar-On R. (2010). Emotional intelligence: An integral part of positive psychology. S. Afr. J. Psychol..

[5] Castillo R., Salguero J.M., Fernández-Berrocal P., Balluerka N. (2013). Effects of an emotional intelligence intervention on aggression and empathy among adolescents. J. Adolesc..

[6] Durlak J.A., Weissberg R.P., Dymnicki A.B., Taylor R.D., Schellinger K.B. (2011). The impact of enhancing students’ social and emotional learning: A metaanalysis of school-based universal interventions. Child. Dev..

[7] Petrides K.V., Frederickson N., Furnham A. (2004). The role of trait emotional intelligence in academic performance and deviant behavior at school. Personal. Individ. Dif..

[8] Petrides K.V., Sangareau Y., Furnham A., Frederickson N. (2006). Trait emotional intelligence and children’s peer relations at school. Sch. Dev..

[9] Russo P.M., Mancini G., Trombini E., Baldaro B., Mavroveli S., Petrides K.V. (2012). Trait emotional intelligence and the big five: A study on Italian children and preadolescents. J. Psychoeduc. Assess..

[10] Costa A., Faria L. (2015). The impact of emotional intelligence on academic achievement: A longitudinal study in Portuguese secondary school. Learn. Individ. Differ..

[11] Duckworth A.L., Seligman M.E.P. (2005). Self-discipline outdoes IQ in predicting academic performance of adolescents. Psychol. Sci..

[12] Panayiotou M., Humphrey N., Wigelsworth M. (2019). An empirical basis for linking social and emotional learning to academic performance. Contemp. Educ. Psychol..

[13] Andrei F., Mancini G., Mazzoni E., Russo P.M., Baldaro B. (2015). Social status and its link with personality dimensions, trait emotional intelligence, and scholastic achievement in children and early adolescents. Learn. Individ. Differ..

[14] Barchard K.A. (2003). Does emotional intelligence assist in the prediction of academic success?. Educ. Psychol. Meas..

[15] Bastian V.A., Burns N.R., Nettelbeck T. (2005). Emotional intelligence predicts life skills, but not as well as personality and cognitive abilities. Pers. Individ. Dif..

[16] Perera H.N., DiGiacomo M. (2013). The relationship of trait emotional intelligence with academic performance: A meta-analytic review. Learn. Individ. Differ..

[17] Qualter P., Gardner K.J., Pope D.J., Hutchinson J.M., Whiteley H.E. (2012). Ability emotional intelligence, trait emotional intelligence, and academic success in British secondary schools: A 5 years longitudinal study. Learn. Individ. Differ..

[18] MacCann C., Jiang Y., Brown L.E., Double K.S., Bucich M., Minbashian A. (2020). Emotional intelligence predicts academic performance: A meta-analysis. Psychol. Bull..

[19] Buenrostro A.E., Valadez M.D., Soltero R., Nava G., Zambrano R., García A. (2012). Inteligencia emocional y rendimiento académico en adolescentes. Rev. Educ. Y Desarro..

[20] Agnoli S., Mancini G., Pozzoli T., Baldaro B., Russo P.M., Surcinelli P. (2012). The interaction between emotional intelligence and cognitive ability in predicting scholastic performance in school-aged children. Pers. Individ. Dif..

[21] Ferrándiz C., Hernández D., Bermejo R., Ferrando M., Sáinz M. (2012). Social and emotional intelligence in childhood and adolescence: Spanish validation of a measurement instrument. Rev. Psicodidáctica.

[22] Ferrando M., Prieto M.D., Almeida L.S., Ferrándiz C., Bermejo R., López-Pina J.A., Fernández M.C. (2011). Trait emotional intelligence and academic performance: Controlling for the effects of IQ, personality, and self-concept. J. Psychoeduc. Assess..

[23] Mavroveli S., Petrides K.V., Sangareau Y., Furnham A. (2009). Exploring the relationships between trait emotional intelligence and objective socio-emotional outcomes in childhood. Br. J. Educ. Psychol..

[24] Mavroveli S., Sánchez-Ruiz M.J. (2011). Trait emotional intelligence influences on academic achievement and school behaviour. Br. J. Educ. Psychol..

[25] Parker J.D., Creque R.E., Barnhart D.L., Harris J.I., Majeski S.A., Wood L.M., Hogan M.J. (2004). Academic achievement in high school: Does emotional intelligence matter?. Pers. Individ. Dif..

[26] Parker J.D., Summerfeldt L.J., Hogan M.J., Majeski S.A. (2004). Emotional intelligence and academic success: Examining the transition from high school to university. Pers. Individ. Dif..

[27] Downey L.A., Mountstephen J., Lloyd J., Hansen K., Stough C. (2008). Emotional intelligence and scholastic achievement in Australian adolescents. Aust. J. Psychol..

[28] Ashcraft M.H. (2002). Math anxiety: Personal, educational, and cognitive consequences. Curr. Dir. Psychol. Sci..

[29] Belmonte V. (2013). Inteligencia Emocional y Creatividad: Factores Predictores del Rendimiento Académico. [Emotional Intelligence and Creativity: Predictors of Academic Performance]. Ph.D. Thesis.

[30] Pérez-González J.C., Cejudo-Prado M.J., Duran-Arias C.R. (2014). Emotional intelligence as a non-cognitive predictor of academic performance. Pers. Individ. Dif..

[31] Bar-On R., Parker J.D.A. (2000). BarOn Emotional Quotient Inventory: Youth Version.

[32] Viguer P., Cantero M.J., Bañuls R. (2017). Enhancing emotional intelligence at school: Evaluation of the effectiveness of a two-year intervention program in Spanish pre-adolescents. Pers. Individ. Dif..

[33] Zeidner M., Roberts R.D., Matthews G. (2002). Can emotional intelligence be schooled? A critical review. Educ. Psychol..

[34] Qualter P., Whiteley H.E., Hutchinson J.M., Pope D.J. (2007). Supporting the development of emotional intelligence competencies to ease the transition from primary to high school. Educ. Psychol. Pract..

